# Supplementary material for: Serological Immune Response Following ChAdOx1 nCoV-19 Vaccine (Covishield®) in Patients with Liver Cirrhosis
Source: Vaccines (Basel). 2022 Oct 30;10(11):1837. doi: 10.3390/vaccines10111837 (PMC9696004; doi:10.3390/vaccines10111837)
Supplement: Supplementary file 1 [file vaccines-10-01837-s001.zip › vaccines-1971956-supplementary.pdf]

Supplementary Table S1: Comparison of clinical characteristics, laboratory values, and vaccine induced antibody response according to age groups

**Variable**	**Age <45 years (n=40)**	**Age ≥45 years (n=91)**	**p value**
Males	32 (80)	61 (67)	0.132
Severity of liver cirrhosis			
Decompensated cirrhosis	17 (40)	35 (38.5)	0.868
Child-Turcott-Pugh score	7 (5-9)	7 (6-8)	
Child-Turcott-Pugh Class			
CTP A	17 (42.5)	35 (38.5)	
CTP B	17 (42.5)	46 (50.5)	0.652
CTP C	6 (15)	10 (11)	
Hemoglobin (g/dL)	12.3 (10.4-14.2)	12.3 (10.8-13.6)	0.805
While cell counts (x1000 mm^3)	4.7 (4.0-6.2)	5.0 (3.8-7.0)	0.275
Platelets counts (x1000 µL)	110 (75-146)	110 (70-140)	0.772
Serum creatinine (mg/dL)	0.8 (0.7-1.0)	0.9 (0.8-1.1)	0.036
Total serum bilirubin (mg/dL)	1.3 (0.7-2.5)	1.2 (0.8-1.9)	0.73
Serum albumin (g/dL)	4.4 (3.6-4.6)	4.0 (3.5-4.4)	0.084
International normalized ratio (INR)	1.4 (1.1-1.7)	1.3 (1.1-1.5)	0.13
Interval between two doses (weeks)	12 (8-22)	12 (11-19)	0.599
Interval between second dose and specimen collection (weeks)	7 (4-11)	8 (5-12)	0.221
Anti-spike antibody titre (U/mL)	4640 (1739-10126)	5521 (1719-9980)	0.812
Neutralizing antibody (%)	95.8 (67.6-98.2)	91.9 (40.7-96.9)	0.093

Categorical data are presented as number and proportions; Numerical data are expressed as median (interquartile range)

Supplementary Table S2: Comparison of clinical characteristics, laboratory values, and vaccine induced antibody response between the groups with viral etiology or non-viral etiology for cirrhosis

**Variable**	**Etiology of cirrhosis**		
	**Viral: HBV/HCV (n=79)**	**Non-viral (n=52)**	**p value**
Males	53 (67)	40 (77)	0.225
Age (years)	50 (43-58)	51 (43-58)	0.94
Severity of liver cirrhosis			
Decompensated cirrhosis	32 (41)	19 (37)	0.649
Child-Turcott-Pugh score	7 (6-8)	7 (6-9)	0.392
Child-Turcott-Pugh Class			
CTP A	35 (44)	17 (33)	
CTP B	37 (47)	26 (50)	0.227
CTP C	7 (9)	9 (17)	
Hemoglobin (g/dL)	12.3 (10.1-13.8)	12.4 (10.8-13.7)	0.38
While cell counts (x1000 mm^3)	5.0 (3.7-6.8)	4.8 (4.0-6.8)	0.884
Platelets counts (x1000 µL)	103 (62-130)	110 (70-140)	0.221
Serum creatinine (mg/dL)	0.9 (0.8-1.1)	0.9 (0.8-1.1)	0.47
Total serum bilirubin (mg/dL)	1.2 (0.8-1.8)	1.3 (0.7-2.4)	0.699
Serum albumin (g/dL)	4.1 (3.5-4.5)	4.1 (3.6-4.6)	0.979
International normalized ratio (INR)	1.2 (1.1-1.4)	1.4 (1.1-1.7)	0.063
Interval between two doses (weeks)	12 (10-22)	12 (8-17)	0.67
Interval between second dose and specimen collection (weeks)	8 (5-12)	7 (6-12)	0.744
Anti-spike antibody titre (U/mL)	5095 (1799-9271)	6012 (1690-11228)	0.571
Neutralizing antibody (%)	89 (49.9-97.3)	94.1 (42.3-97.8)	0.35

Categorical data are presented as number and proportions; Numerical data are expressed as median (interquartile range)
